# A community-based intervention (Young SMILES) to improve the health-related quality of life of children and young people of parents with serious mental illness: randomised feasibility protocol

**DOI:** 10.1186/s13063-018-2935-6

**Published:** 2018-10-11

**Authors:** Judith Gellatly, Penny Bee, Lina Gega, Peter Bower, Diane Hunter, Paul Stewart, Nicky Stanley, Rachel Calam, Kim Holt, Miranda Wolpert, Simon Douglas, Jonathan Green, Adekeye Kolade, Craig Callender, Kathryn M Abel

**Affiliations:** 1 Centre for Mental Health and Safety and Centre for Women’s Mental Health, Division of Psychology and Mental Health, Faculty of Biology, Medicine and Health, Manchester Academic Health Science Centre, The University of Manchester, Manchester, UK; 2 Division of Nursing, Midwifery and Social Work, Faculty of Biology, Medicine and Health, Manchester Academic Health Science Centre, The University of Manchester, Manchester, UK; 3 Department of Health Sciences, University of York, York, UK; 4 Division of Population Health, Health Services Research & Primary Care, Manchester Academic Health Science Centre, The University of Manchester, Manchester, UK; 5 NSPCC, Weston House, London, UK; 6 NSPCC, Leeds, UK; 7 School of Social Work, Care and Community, University of Central Lancashire, Preston, UK; 8 School of Psychological Sciences, Faculty of Biology, Medicine and Health, Manchester Academic Health Science Centre, The University of Manchester, Manchester, UK; 9 Department of Social Work, Education & Community Wellbeing, Northumbria University, Newcastle, UK; 10 Anna Freud Centre, London, UK; 11 Research and Development, Northumberland, Tyne and Wear NHS Foundation Trust, St. Nicholas Hospital, Newcastle upon Tyne, UK; 12 School of Biological Sciences, Division of Neuroscience & Experimental Psychology, Manchester Academic Health Science Centre, The University of Manchester, Manchester, UK; 13 Northumberland, Tyne and Wear NHS Foundation Trust, St. Nicholas Hospital, Newcastle upon Tyne, UK

**Keywords:** Parental mental illness, children, young people, health-related quality of life, intervention, feasibility, qualitative

## Abstract

**Background:**

Children and young people of parents with mental illness (COPMI) are at risk of poor mental, physical and emotional health, which can persist into adulthood. They also experience poorer social outcomes and wellbeing as well as poorer quality of life than their peers with ‘healthy’ parents. The needs of COPMI are likely to be significant; however, their prevalence is unknown, although estimates suggest over 60% of adults with a serious mental illness have children. Many receive little or no support and remain ‘hidden’, stigmatised or do not regard themselves as ‘in need’. Recent UK policies have identified supporting COPMI as a key priority, but this alone is insufficient and health-related quality of life has been neglected as an outcome.

**Methods/design:**

An age-appropriate standardised intervention for COPMI, called Young SMILES, was developed in collaboration with service users, National Health Service (NHS) and non-NHS stakeholders in our previous work. This protocol describes a randomised feasibility trial comparing Young SMILES with usual care, involving 60 families that will be identified through third sector organisations and NHS services, and recruited and randomised on a 1:1 basis to receive Young SMILES or usual care. Outcomes of the feasibility trial are rates of recruitment, follow-up and withdrawals, intervention uptake, and engagement. The optimal child-reported outcomes will also be determined alongside the assessment of resource use. A qualitative evaluation conducted at 3-months will explore the experiences and views of children and young people as well as parents accessing the intervention and the facilitators delivering the intervention.

**Discussion:**

This paper details the rationale, design, training and recruitment methods for a feasibility study to inform the design and effective implementation of a larger scale randomised controlled trial of Young SMILES.

**Trial Registration:**

ISRCTN36865046, registered 18 December 2015.

**Electronic supplementary material:**

The online version of this article (10.1186/s13063-018-2935-6) contains supplementary material, which is available to authorized users.

## Background

Current estimates suggest that over 60% of parents with a serious mental illness (SMI) live with one or more children or young people (CYP) under the age of 18 [1]. Evidence indicates that CYP living, or in regular contact with a parent with SMI can be vulnerable to poorer mental and physical health, behavioural, social and educational difficulties, as well as maltreatment and neglect in comparison to other CYP [2]. The effects can be long-lasting, with many at increased risk of socio-occupational dysfunction, psychiatric morbidity, alcohol or substance misuse, and premature death [3] in adulthood. CYP of parents with SMI are said to have a 50% chance of developing a mental health problem, with a 32% probability of developing a SMI [4]. The problems arise not only because parents with SMI find it difficult to manage their role as carers, but also because they are often coping with multiple deprivation and have ongoing concerns about their children being moved to out-of-home care [5].

Many of the difficulties that CYP of parents with mental illness (COPMI) face can lead to poor health-related quality of life (HRQoL). Several UK policy initiatives offer perspectives on CYP’s social and emotional wellbeing, including Social and Emotional Wellbeing for Children and Young People [6], Think Child, Think Parent, Think family [7], the Children’s and Young People’s Strategy in Northern Ireland [8], the Getting it Right for Every Child approach in Scotland [9], Working with Troubled Families [10], and the Children’s and Young People’s Strategy [11]. Collectively, these policies clarify the responsibility and supportive nature that services have in ensuring the safeguarding and wellbeing of CYP who have parents experiencing mental illness; further, the importance of ensuring their needs, in addition to those of their parent, is explicitly identified in a timely and appropriate manner to ensure they are addressed effectively.

The importance of addressing the needs of COPMI is also identified internationally, for example, through the European Union’s Child and Adolescent Mental Health in Enlarged Europe CAMHEE initiative [12, 13], the Children of Parents with a Mental Illness Framework for Mental Health Services 2010–2015 in Australia [14] and the Child Welfare Act in Finland [15]. Five broad quality of life domains are shared between these initiatives and are highlighted within the current agenda for CYP as (1) health, (2) safety, (3) economic well-being, (4) enjoyment and achievement, and (5) positive societal contribution.

Despite recognition of the support needed for COPMI, there is a significant lack of reliable evidence for the effectiveness of any evidence-based interventions for this population, particularly within the UK National Health Service (NHS) [16]. It can be challenging for parents with SMI and their children to access the support they need. Integrated care for COPMI is complex because NHS adult mental health, Child and Adolescent Mental Health Services (CAMHS), social care, and child protection services are located and managed separately; this can mean that CYP ‘fall through the gaps’ between different health and social care providers. Furthermore, because services may not consider the CYP to be ‘at risk’, many services have little to offer [17]. Added to this, many CYP may not identify themselves as having a ‘need’ for intervention. Those who do may have concerns about the stigma of mental health problems or worry about the consequences of asking for help, for example, on their parents [18].

One of the biggest challenges is identifying when and how to support COPMI effectively in a non-stigmatising and accessible way. Our consultation work with CYP [19] expressed the need for interventions to improve their coping skills and mental health literacy. The earlier phase of this study, involving focus groups and interviews with parents with mental illness, highlighted that CYP and parents desired mental health literacy, and that communication and problem-solving skills should be the driving principles underlying each of the interventions. These views were mirrored by professionals working in the NHS and third sector organisations. A more recent mixed-methods study additionally supports these findings [20]. It is thought that a significant “*paradigm shift is required at all levels of service development, delivery and policy*” [21] to enhance the lives of these CYP and their families.

Since late 2011, the National Society for the Prevention of Cruelty for Children (NSPCC) has been providing and evaluating an intervention called Family SMILES (Simplifying Mental Illness + Life Enhancement Skills) for families with parental mental illness, particularly those (but not exclusively) where CYP were assessed as at risk of abuse or neglect. Family SMILES aims to improve CYP’s self-esteem, enhance parents’ protective abilities and improve parent–child relationships in CYP aged 8–13 years. The NSPCC’s preliminary evaluation of Family SMILES was a single-group, pre-test/post-test measurement of change on self-reported outcomes of strengths and difficulties, self-esteem and child abuse risk [22]. Of importance for the present study, the evaluation highlighted the potential benefits (1) for children (increased social functioning and confidence, reduced social isolation and reduced blame associated with parental illness); (2) for parents (less distress and unhappiness, shift of thinking from own need to children’s needs); and (3) for families (more relaxed atmosphere, openness about parental mental health, empathy between child and parent, shared responsibilities). There is significant potential for this intervention to be manualised, extended and enhanced with respect to supporting a wider age range and explicitly reaching out to CYP who may not have been identified as ‘at risk’ and to be implemented within an NHS context. Rather than the focus being on the whole family, there is the opportunity to focus specifically on enhancing the children’s quality of life.

### Aims and objectives

The aim of the study is to evaluate a co-developed (with stakeholders), standardised and community-based intervention for COPMI to improve their HRQoL.

The primary objectives are:

- Using a feasibility randomised controlled trial (RCT) comparing the intervention developed in our earlier work with usual care to determine uptake, adherence and follow-up rates.
- To determine, from a battery of outcome measures, the most appropriate primary outcomes with which to assess any effects of the intervention over time, considering the areas identified as important by the stakeholders.
- To develop and pilot a data collection tool relevant to family resource use over time.
- To determine if the intervention is acceptable to COPMI, their parents and the practitioners delivering the intervention.
- To establish if the intervention can be implemented successfully within third sector and NHS settings.

## Methods/Design

### Trial design

This study is a feasibility RCT designed to assess the methodology proposed for the conduct of a future definitive RCT. It will evaluate the Young SMILES intervention in comparison to usual care alone. Families will be randomised to (1) Young SMILES or (2) usual care. Those allocated to Young SMILES will also be able to access any usual care services should they wish or require to do so. Families will be asked to complete outcome measures at baseline, 3-months, 6-months and 12-months following the baseline assessment. While this is a feasibility study and long-term follow-ups are not normally necessary, one of the findings within the Bee et al. review [16] was that no one carried out long-term outcomes for CYP and thus this was regarded as a useful opportunity to test the viability of doing so.

**Fig. 1:**
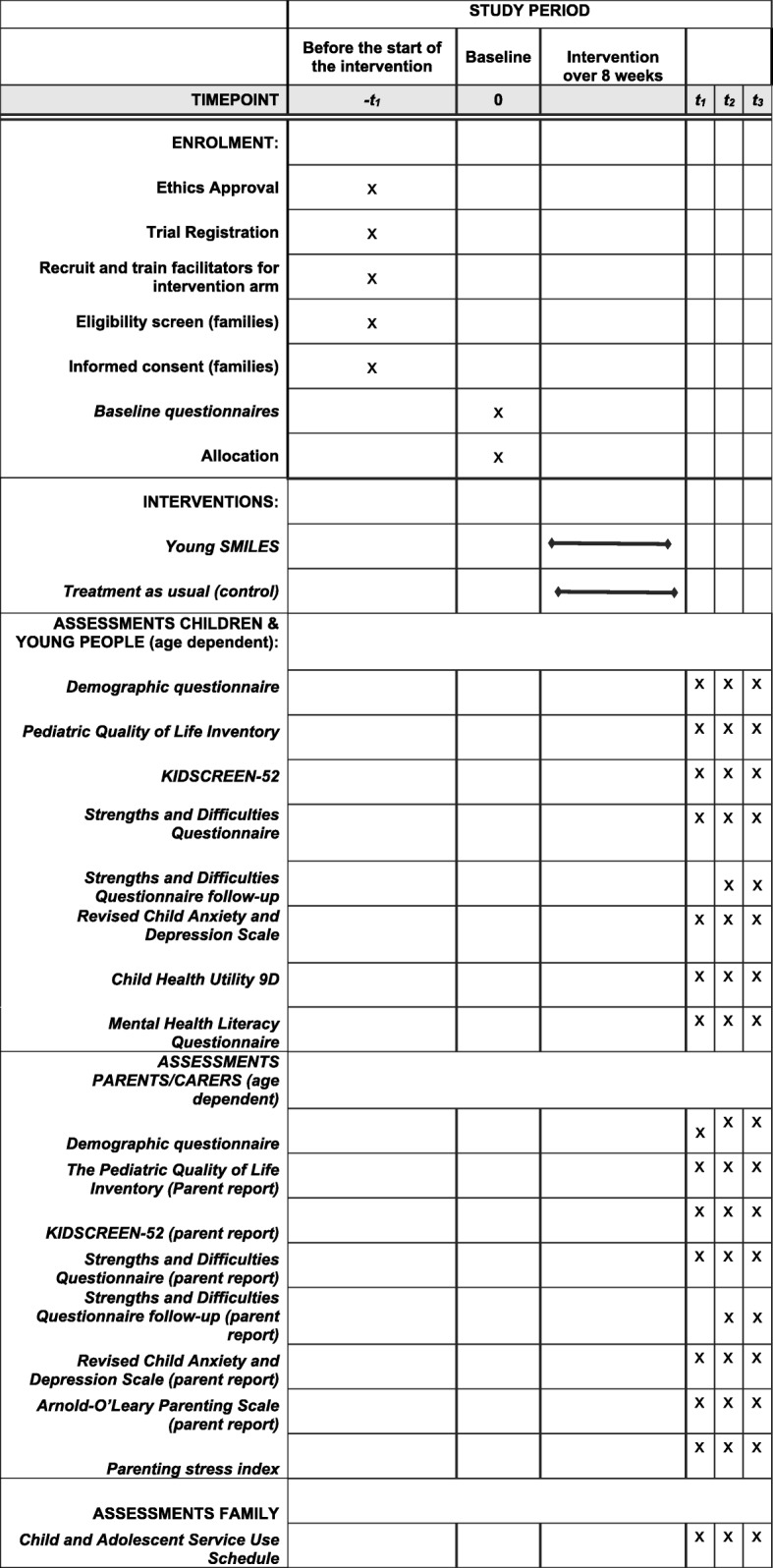
SPIRIT. Schedule of enrolment, assessment and interventions

A SPIRIT checklist is attached as an Additional file 1 and Fig. 1 shows the schedule of enrolment, assessment and interventions (SPIRIT).

### Settings and participants

A total of 60 families who did not participate in the development of the intervention will be recruited. They will be recruited via new and existing referral pathways from an NHS Foundation Trust site, including CAMHS and Adult Mental Health services, and third sector organisations who work with COPMI.

### Inclusion/exclusion criteria

#### Inclusion

1. CYP, aged 6–16 years, of parents diagnosed with SMI (e.g. schizophrenia, bipolar disorder, psychosis).
2. Parents/carers with SMI and their partners (who may or may not have any mental health problems). The focus of our project is the CYP and their outcomes, rather than the parents. Therefore, we do not intend to carry out full clinical interviews with the parents and report diagnostic codes. We shall accept the primary and secondary diagnoses reported by a key health professional, such as a general practitioner (GP), care coordinator or key worker, as most of these parents are likely to receive secondary care or be monitored in primary care. This can be gleaned during referral into the study or, in the case of self-referral by the parent, we shall obtain the diagnosis by contacting the parent’s appropriate care coordinator, e.g. GP or community psychiatric nurse, following the parent’s permission to do so.
3. The CYP must have at least 10 h of carer with/carer with SMI each week (the CYP does not necessarily have to live with a mentally ill parent).
4. The parents/carers understand the purpose and remit of the intervention for themselves and their children and consent to their and their child’s attendance and completion of outcome measures and interviews.
5. The CYP understands the purpose and remit of the intervention and consent, if competent, to attend group sessions and complete outcome measures and interviews.
6. The parents and CYP can understand basic written and verbal information in English.
7. The CYP has some awareness of the parent’s mental illness, confirmed by the parent and/or the appropriate care coordinator. The CYP does not necessarily have to be able to name the mental illness that is being experienced but provide indication that they know their parent is experiencing difficulties or identify symptoms related to the illness. If the CYP has no awareness of the parent’s illness, we shall discuss how the parent and care coordinator can prepare the CYP before they start group work.

#### Exclusion

1. CYP of parents diagnosed with common mental disorder (e.g. mild to moderate depression) or with primary substance misuse, rather than with a SMI as defined in inclusion criterion 1 above.
2. The CYP has significant cognitive impairment or a learning disability or major mental illness or behavioural or communication problems (as verified by their GP or other health professionals involved in the family’s care) and/or do not understand basic verbal or written information in English, which would make it impossible or unsafe for them to participate in group work.
3. The parent is extremely unwell at the time of eligibility assessment or has severe communication disabilities or cannot understand basic verbal or written English, which would make it difficult or unsafe for them to participate in standardised group or individual work.
4. The CYP has already participated in Family SMILES.

### Intervention design

#### Comparator – usual care

The control group will be usual care, defined as access to any services or resources to which CYP and their families would usually be referred or have access. Participation in the trial will not preclude access to these.

#### Intervention – Young SMILES

Young SMILES is a service for CYP (aged 6–16) whose parents live with SMI, aiming specifically to improve the HRQoL of CYP. The intervention was co-developed in the earlier phase of this research via consultation with stakeholders (CYP of parents with serious mental illness, parents and practitioners from third sectors and NHS organisations). Young SMILES is an 8-week group programme (4–6 CYP per group) split into two age bands, namely 6–11 and 12–16. At week 4, five parallel sessions are offered to the parent or significant other adult. CYP and parent sessions last for 2 hours, which includes time for a short break and refreshments during and after the group. Where possible, the sessions will be held in a non-stigmatising venue such as a community location rather than a hospital clinic or school.

Sessions will be facilitated by two trained practitioners. The expertise and skills of the facilitators is an invaluable aspect of the Young SMILES intervention. Groups will be delivered in an NHS organisation using a co-delivery model where one facilitator is from the NHS (family therapy background) and the other is from a third sector organisation. Third sector organisation facilitators will have a variety of professional backgrounds such as social workers, children’s service practitioners, psychologists and occupational therapists.

Intervention facilitators will be provided with a 3-day training session. A training manual for facilitators was developed as part of the development phase which provides an overview of the feasibility trial and development of Young SMILES. It additionally incorporates structured detail on service delivery and intervention delivery, providing an on-going guide/reference for practitioners who deliver the service and manual to support trainers to ‘teach’ practitioners the aspects of the model and how it should be delivered.

##### CYP group sessions

Each session has specific aims and objectives. A brief outline of the session objectives is shown in Table 1.

**Table 1:** Brief outline of children and young people group sessions

Session	Objectives
Session 1: Welcome to Young SMILES	Understand the aim of the group and introduce key themes, e.g. the fictitious family
Session 2: All about me	Understand a sense of self and identify personal strengths and qualities
Session 3: What happens in my family?	Understand mental illness and the impact it can have on a young person’s family
Session 4: Things we worry about	Identify the sources of feelings and understand healthy and unhealthy responses to them
Session 5: Our world	Identify key sources of stress and the building blocks needed for a foundation of feeling good
Session 6: Where do I go when I need help?	Identify support networks and learn how to access help from professionals
Session 7: Enjoying being me	Understand personal strengths and aspirations; recognise which aspirations they can shape
Session 8: Moving on together	Celebrate progress, consolidate relationships and plan for the future

The aims and objectives for both age groups (6–11 and 12–16) will stay consistent. The activities and communication style used to achieve these, however, may differ. Identifying the abilities of children in the group and modifying the activities to match these will be an important role of the facilitators; this will be discussed and addressed within the training provided.

Themes that cut across all sessions will include mental health literacy, communication and problem-solving skills. Throughout the life of the group, the outcomes of each session will be facilitated through the creation of an ‘Imaginary Family’ for younger children (made from cardboard cut-outs/cartoon characters created electronically and printed) and a ‘Graffiti Wall Family’ for older children. A typical session format will include a ‘getting to know you’ (first session)/‘welcome back’ and ‘check-in’ on previous week, ‘ice-breaker’ activity, activities based on the session aims and snack time. A ‘Weekly Challenge’ at the end of each session will be included to orientate and tether COPMI to the next session in order to optimise engagement.

##### Parent/carer group sessions

The content and focus of each session will be determined during the CYP session in the previous week to ensure that the sessions are driven by the CYP to meet their needs. A brief outline of the session objectives is shown in Table 2.

**Table 2:** Brief outline of parent/carer group sessions

Session	Objectives
Session 1: Welcome to Young SMILES	Understand the purpose of the group and start to share family information safely
Session 2: What our children do well	Develop insights into how parents/carers can (and do) encourage and support their children to do well and feel good about themselves
Session 3: What our children worry about	Identify sources of stress in their children and understand healthy and unhealthy responses
Session 4: How we support our children	Identify obstacles to successful family communication and identify support networks
Session 5: Moving on together	Celebrate progress, consolidate relationships and plan for the future

The final ‘Moving on together’ session provides CYP and parents with the opportunity to review progress. The CYP and parents come together for the last few activities, which focus on hopes and fears, achievements, and moving forward.

The activities for each session are standardised, but not prescriptive. As long as they meet the overall objectives of the sessions, and are consistent with the ethos of the Young SMILES Programme in general, the facilitator may choose to use alternative activities that they feel may be more appropriate and effective at the time and for the individuals in the group.

#### Outcome assessment

A summary of the primary and secondary outcomes and tools that will be used can be found in Table 3. Where an age appropriate version has not been developed, only the parent report version will be used. Despite measures being available for differing ages, some CYP may experience difficulties completing some or all of them. Researchers will be responsive to the CYP’s needs and will support completion of the self-report measures as and when required.

**Table 3:** Primary and secondary outcome measures

Outcome	Measured by/using
Primary outcome	
Health-related quality of life	The Pediatric Quality of Life Inventory and KIDSCREEN
Secondary outcomes	
Child psychopathology and prosocial behaviour	Strengths and Difficulties Questionnaire
Symptoms of common mental health problems	Revised Child Anxiety and Depression Scale
Knowledge and perceptions about serious mental illness (mental health literacy)	Mental Health Literacy Questionnaire
Parenting competencies	Arnold-O’Leary Parenting Scale
Degree and cause of stress in a parent–child relationship	Parenting Stress Index/Short Form
Incremental health gain in quality-adjusted life years	Child Health Utility 9D
Resource use	Child and Adolescent Service Use Schedule
Children and young people, parent and facilitator acceptability	Qualitative interviews

#### Primary outcome

For the purposes of the original funding proposal, the primary outcome is CYP’s HRQoL during the feasibility study. Two standardised questionnaires measure child/adolescent HRQoL, as indicated below.

##### Pediatric Quality of Life Inventory

The Pediatric Quality of Life Inventory [23] measures HRQoL in CYP with and without health conditions. It is a reliable tool that measures core dimensions of health as defined by the World Health Organization and in addition to role (school) functioning. It measures physical functioning (8 items), emotional functioning (5 items), social functioning (5 items), and school functioning (5 items). The measure has been validated for CYP aged 2–18 and a parent report is also available. For children under 5 years old, the parent report will be used as a proxy. Each item uses a 4-point scale, ranging from ‘never’ to ‘almost always’ (excluding the 5–7 child self-report, which uses a developmentally-appropriate 3-point scale, ranging from ‘not at all’ to ‘a lot’). A 0–100 score can be calculated as a mean of the items, with higher scores indicating better HRQoL.

##### KIDSCREEN-52

KIDSCREEN [24] measures 10 HRQoL dimensions, including Physical (5 items), Psychological Well-being (6 items), Moods and Emotions (7 items), Self-Perception (5 items), Autonomy (5 items), Parent Relations and Home Life (6 items), Social Support and Peers (6 items), School Environment (6 items), Social Acceptance (Bullying) (3 items), and Financial Resources (3 items). It has been validated for CYP aged 8–18 and a parent report is also available. For children under 8 years old, the parent report will be used as a proxy. Each item uses a 5-point scale, ranging from ‘never’ to ‘always’; higher scores indicate better HRQoL.

#### Secondary outcomes

Secondary outcomes were determined via consultation with experts and review of existing literature focusing on COPMI populations. Three measures are to be completed by CYP and parents, one by CYP only and two by parents only, as indicated below.

##### Child psychopathology and prosocial behaviour (CYP and parents)

Child psychology and prosocial behaviour was measured using the Strengths and Difficulties Questionnaire [25], which is routinely used by the Children and Young People's Improving Access to Psychological Therapies programme as a primary outcome measure and was one of the main assessments in the NSPCC’s Family SMILES evaluation. It will therefore allow maximum comparison with other services and research studies. The tool is a brief emotional and behavioural screening questionnaire for 3–16 year olds, measuring 25 items on five subscales in the following domains: Emotional Symptoms (5 items), Conduct Problems (5 items), Hyperactivity/Inattention, Peer Relationships (5 items), Prosocial Behaviour (5 items), plus an overall total score of difficulties (which excludes the prosocial score). Each item uses a 3-point scale with responses as ‘not true’, ‘somewhat true’ and ‘certainly true’. The level of strengths and difficulties indicated by the score is divided into three categories as ‘normal’ (0–13), ‘borderline’ (14–16) and ‘abnormal’ (17–40).

##### Incremental health gain in quality (CYP and parents)

Quality-adjusted life years (QALYs) will be estimated using the Child Health Utility 9D (CHU-9D). To inform a future economic evaluation, we shall use the CHU-9D [26], which has been validated for CYP aged 7–17 to estimate incremental health gain in QALYs. It uses a descriptive system and a set of preference weights. Utility values are assigned to each health state that is described by the descriptive system, which can be calculated on the basis of QALYs for use in cost utility analysis. The descriptive system consists of nine dimensions, namely Worried, Sad, Pain, Tired, Annoyed, Schoolwork/Homework, Sleep, Daily Routine, and Activities. Each dimension has five levels. The measure is preference-based and therefore suitable for use in paediatric economic evaluation.

##### Symptoms of common mental health problems (CYP and parents)

Symptoms of common mental health problems will be captured using the Revised Child Anxiety and Depression Scale [27], which is a 47-item questionnaire that measures the reported frequency of anxiety and low mood. The measure has been validated for CYP aged 8–18. A parent report is also available. It measures a total score for anxiety and low mood, corresponding to the DSM diagnoses of separation anxiety, social phobia, generalised anxiety, panic, obsessive compulsive, and major depressive disorder. Each item uses a 4-point scale and is assigned with a 0–3 numerical value, with responses as ‘never’ (0 points), ‘sometimes’ (1 point), ‘often’ (2 points) and ‘always’ (3 points). Scores are calculated by converting the raw scores into the corresponding T-scores. While a T-score of 65 or higher indicates the borderline clinical threshold, a T-score of 70 or higher indicates above the clinical threshold for anxiety and depression.

##### Children’s knowledge and perceptions about SMI (mental health literacy) (CYP only)

We shall use the Mental Health Literacy Questionnaire [28, 29] to assess the CYP’s knowledge and perceptions about SMI (mental health literacy) and their problem-solving skills. The measure has 48-items and is divided into four sections as (1) sociodemographic information; (2) knowledge about mental health problems (32-items); (3) first aid skills and help seeking (10-items); and (4) self-help strategies (6-items). Each item has a 5-point Likert scale, ranging from ‘strongly disagree’ (1 point) to ‘strongly agree’ (5 points). The last item has a multiple-choice option, asking CYP to identify mental health problems from a list of 11 health problems. Higher scores indicate higher levels of mental health literacy.

##### Parenting competencies (parents only)

We shall measure parenting skills and child-parent relationships using the Arnold-O’Leary parenting scale [30]. This tool is a 30-item questionnaire that measures parents on three factors, namely Laxness (11 items), Over-Reactivity (10 items) and Verbosity (7 items). Four more items are not on a factor. Each item has a 7-point Likert scale (7 being the ‘ineffective’ end of the item). Total scores are calculated as an average of the responses on all items. Factor scores are calculated as an average of the responses on the items in that factor. Higher scores indicate higher degrees of parental laxness, over-reactivity and verbosity.

##### The degree and cause of stress in a parent–child relationship (parents only)

The degree and cause of stress in a parent–child relationship was measured using the Parenting Stress Index/Short Form [31]. This tool has 36-items that are divided into three categories of stress, namely Parental Distress, Parent-Child Dysfunctional Interaction and Difficult Child, which combine to form a Total Stress scale. Scores are calculated by converting raw scores into percentile and T-scores. To be in the ‘normal range’ of parental stress, parents have to be in between 16th to 84th percentile; to be in the ‘high range’, parents have to be in between the 85th to 89th percentile; and to be in the ‘clinically significant range’, parents had to be in the 90th percentile or above. A Defensive Responding score is clinically significant when the raw score is 10 or lower.

### Resource use

Resource use will be assessed using the Child & Adolescent Service Use Schedule designed in previous research [32], adapted for the purpose of the current trial. It will collect information on receipt of care and services by each family in relation to CYP needs and services from the NHS, social care, education, voluntary and third sector organisations. The cost of this resource use will be primarily calculated from the NHS and Personal Social Services perspective, with a secondary wider societal perspective estimate also calculated in light of the societal/community context of the intervention.

### Acceptability

The success of intervention implementation heavily relies on its acceptance by those in receipt of and those delivering an intervention. A qualitative evaluation of the intervention will be conducted via interviews or focus groups with CYP, parents/carers and practitioners post intervention, at 3 months. All individuals who accessed or delivered Young SMILES will be invited to participate, including those who leave treatment early. All individuals who express an interest in taking part will be given the option of taking part in an individual interview or focus group. If participation in a focus group is selected, in order to ensure participants feel at ease, group composition will be considered and the different stakeholders (CYP of differing age bands, parents and professionals) will take part in separate group discussions.

Semi-structured interview schedules will be prepared to guide the discussions. Interviews with CYP and parents/carers will focus on identifying barriers and facilitators to attending, what they liked and disliked, and if they think Young SMILES has helped their family. Discussions with children will follow the semi-structured interview schedule but will additionally use child-centred approaches such as drawing or inclusion of visual resources to aid understanding and enhance data collection. Practitioner interviews will perceptively explore what factors may facilitate or hinder the implementation of Young SMILES.

At baseline, we will additionally ask the parent or carer and the child to complete a demographic questionnaire.

### Sample size

The proposed sample size of 60 randomised families (30 per group as recommended for pilot studies [33]) is adequate to facilitate the main aims of the study such as establishing feasibility and informing a future power calculation. Formal power calculations are not appropriate for this feasibility study.

We aim to run a minimum of three sets of child and adolescent groups and three parallel parent groups over the three recruitment sites during the recruitment period. We shall offer the parenting element of the intervention to both the ‘ill’ and the ‘well’ parent /carer (if they both wish to participate) or a significant family member, and we shall offer child- and adolescent-centred work to all eligible CYP within each family.

### Recruitment

We will utilise existing successful NSPCC Family SMILES referral pathways such as identifying families with the assistance of practitioners working in CYP services, CAMHS, Adult Mental Health services and education services. We will broaden the diversity of referral sources by working closely with NHS practitioners and services. We will apply for adoption by the National Institute for Health Research’s Clinical Research Network to support recruitment and the identification of suitable families via GP registers, Community Mental Health and Inpatient Teams, and rehabilitation units. Posters and flyers will be placed in appropriate NHS third sector organisations and schools, and practitioners working in the community will be informed of the study to assist with the generation of direct opportunistic referrals.

Recruiting gatekeepers will provide potential families with a study information pack including an invitation letter, information sheet and consent-to-contact form. Separate packs containing the same information will be provided to CYP and parents. If a family are interested in participating, they can return the consent-to-contact form, detailing their preferred method(s) of contact, directly back to the research team in the freepost envelope also included in the information pack. Families who are informed of the study by a practitioner or support worker will also have the opportunity to ask them to complete a contact form on their behalf and return it securely to the research team. Following receipt of a consent-to-contact form, the research team will contact the family, identify if the family are suitable based on the inclusion/exclusion criteria outlined previously and confirm that they are still interested in taking part. If eligible families will be informed that they will be contacted at a later stage to arrange and complete a face-to-face baseline interview at a convenient time and place. The timing of the baseline interviews will be dependent on the research team receiving enough referrals of the same age band at one site. Families will be contacted by telephone fortnightly during the waiting period by the research team to be informed of progress and to provide them the opportunity to answer any additional questions. A one-off home visit to discuss the study will also be offered.

At the face-to-face interview, the family will be given the opportunity to ask any further questions and written consent will be taken. As the CYP will be 16 years old or younger, they will be asked to give assent and consent from their parents/carer will be obtained in support of their child’s participation. Separate consent will be obtained for parents/carers who are also participating. It will be made clear to all family members that they can withdraw from the study at any point, without detriment to their care. If a parent wishes to withdraw, their child/children can still participate with their consent. If the parent has more than one child, all children in the family will be eligible to participate, but are not required to do so. If siblings are in the same age band, they will attend different groups.

After obtaining consent, family members wishing to participate in the study will be asked to complete the baseline measure booklet. All measures are standardised and have been designed as self-complete; however, the researcher will be able to offer any assistance when and if required. Outcome measure booklets will be age-dependent according to the age-range for which the measures have been designed. Families will receive a total of £50 shopping vouchers over the data collection time points for their participation in the study.

During the baseline and subsequent follow-up visits, trial researchers will identify if any adverse events have occurred and report as per agreed procedures. An identified member of the Trial Steering Committee (TSC) will monitor participant safety within the trial and will be responsible for reviewing any serious adverse events occurring as part of the trial.

### Randomisation

Following agreement to participate via written consent and completion of baseline outcome measures, families will be randomised on a 1:1 ratio either to Young SMILES or to usual care using a central, independent, free web-based system [34].

### Blinding

Randomisation will be conducted by the study co-ordinator to maintain blinding of the statistician and research team to reduce detection bias. To facilitate blinding, we will (1) ensure that families know that they can contact the study coordinator should they have any queries about their allocation or the Young SMILES intervention arrangements and (2) the researcher conducting the data collection visits will remind families to refrain from revealing their allocation to them during follow-up visits. Should a researcher become aware of any allocations, this will be recorded and reported.

### Analysis

#### Statistical analysis

##### Determine uptake, adherence and follow-up rates

The main focus will be on tabulated and associated graphical summaries of the key indicators of success of the study, including recruitment and participant flow. We shall report data in line with the Consolidated Standards of Reporting Trials (CONSORT) statement [35]. We shall also report the numbers of participants who drop out from the intervention, withdraw their consent and do not provide follow-up outcome data.

##### Intervention effectiveness

In order to inform a future definitive trial, we shall use standard linear regression to examine change in our HRQoL measures, Pediatric Quality of Life Inventory and KIDSCREEN, adjusting for baseline scores and the sex of the child. The presentation of the analysis will focus on point estimates and associated 95% confidence intervals. After obtaining point estimates of intervention effect and measure of variability of the outcomes, we will be able to design a definitive trial. As this is a feasibility trial, the subgroup analyses will not be conducted but will be addressed should a subsequent large scale trial be conducted.

If multiple children and parents are recruited from the same family, for the main analysis, we will identify an index child and parent for inclusion in the analysis from whom data will be collected. Other sibling(s) will still be offered the opportunity to attend Young SMILES (if their family is randomised to the intervention group). We shall ask parents to nominate the index child. We will discuss with parents which child should be ‘nominated’ as the index child. Parents will not be party to any data collected from their child/children. Since outcome data is collected for all children and parents, in a secondary analysis, we shall include all available data and use robust standard errors to account for clustering within families. Within-cluster variation due to the group intervention will be estimated for the main clinical outcomes in order to inform plausible intra-cluster correlations for sample size calculation of the main cluster RCT.

#### Economic analysis

A prospective economic evaluation will be rehearsed to develop and refine methods for a subsequent definitive RCT.

##### Resource use data collection tool

The main focus will be on how to accurately identify, quantify and value costs of delivering Young SMILES as an addition to usual care, and its potential resource implications for the NHS, versus usual care alone, during our follow-up period. The Child & Adolescent Service Use Schedule tool has been adapted for use in the context of Young SMILES to capture resource use accurately by families in relation to children’s needs and services across the NHS, social care, and voluntary/third sector organisations. We shall identify appropriate unit costs for each area of resource use, which will be obtained from a combination of local and national sources. We ill then assess the feasibility of this measure for use in a future economic evaluation. The corresponding preference weights will be applied to CHU-9D scores to calculate QALYs between baseline and follow-up. Completion rates of the questionnaire will be assessed, along with correlations with the primary and secondary outcome measures, and changes in these measures over time. We shall rehearse the methods to estimate an incremental cost-effectiveness ratio for Young SMILES plus usual care versus usual care alone, in terms of HRQoL years gained.

#### Qualitative analysis

Acceptability interviews will be transcribed verbatim and data will be analysed using Framework analysis [36], a popular way of analysing primary qualitative data pertaining to healthcare practices with policy relevance [37]. Framework analysis permits both deductive and inductive coding, enabling potentially important themes or concepts identified a priori to be combined with additional emerging themes. Data coding will be undertaken by two researchers independently. An initial coding framework will be developed and regular discussions will take place to discuss emerging codes and ensure they are grounded in the original data. Any discrepancies will be discussed and resolved with the wider research team. Codes in each interview will be examined across individual transcripts as well as across the entire dataset and allocated to the framework. Using aspects of the constant comparative method of analysis, broader categories using linking codes will be developed across interviews. Data will be interpreted and analysed within the framework to structure participants’ views about the Young SMILES intervention. The final coding framework will be presented to the wider research team and project steering committee to confirm coherence and conceptual relevance. Data will be analysed without knowledge of trial outcomes in order to avoid biasing, as recommended by the MRC process evaluation model [38].

### Ethics, Health Research Authority (HRA) and research governance approval

The study received a favourable ethical opinion from the National Research Ethics Committee on May 30, 2017 (East of England - Cambridge South REC, Ref 17/EE/0175; IRAS Project ID 215168). HRA approval was received on June 1, 2017. Research governance approval for all participating sites has been sought and obtained.

The study will be conducted in accordance with the UK Policy Framework for Health and Social Care Research [39] and adhere to principles of the Helsinki Declaration [40]. All information collected during the course of the study will be stored securely, anonymised and secured off-site following completion of the study for a period of at least 5 years in accordance with the University of Manchester’s policy on storage of personal data and in line with current data protection legislation.

### Trial governance

The Trial Management Group, composed of the Chief Investigator, grant co-applicants, recruitment site representatives and research team members, will provide overall management of the study.

Independent supervision of the trial will be conducted by members of the TSC. The TSC will be composed of an independent chair and eight other members, including a lay member who has lived experience of being a parent experiencing SMI. The Trial Manager along with the Chief Investigator will attend the TSC meetings.

### Forecast execution dates

Phase I of the study, which included the development of the intervention and obtaining ethical approval for the feasibility trial, was complete in June 2017. Recruitment of participants via NHS lists and third sector referral pathways commenced in July 2017 and will be complete in May 2018. The first intervention group took place in January 2018, additional groups will continue to commence until the end of June 2018. Qualitative interviews will start in March 2018, with data analysis running alongside data collection. Analysis of quantitative data will commence July 2018.

### Dissemination

We shall publish the results in a variety of high quality, peer reviewed, scientific journals for different professional groups, including psychiatry, nursing, social work, psychology, psychotherapy and education. We shall present at national and international conferences for service users, non-governmental organisations, policy-makers, and those responsible for service design and commissioning. We shall arrange a stakeholder conference to discuss our findings as well as publish a lay summary of findings on the website and through our partner networks in NSPCC, Barnardo’s and other third sector organisations.

We shall work with the NSPCC as part of our dissemination plan to draw up an open IP licence agreement that will enable the large-scale use of Young SMILES on a not-for-profit basis. This is an important pathway to research impact. We shall work with national and local service user groups and agencies, such as Rethink Mental Illness and Young Carers, to communicate the findings of our research, drawing on these organisations’ existing partnerships and knowledge transfer programmes.

We shall approach universities and other organisations who offer training and development courses to professionals working with mentally ill people (such as social workers and community psychiatric nurses) so that they become aware of the intervention and explore the most appropriate ways of offering Young SMILES as optional skills training.

### Protocol changes

Since the trial commenced, a number of protocol changes have been made. These are detailed in Table 4. Applications to the Research Ethics Committee (REC)/HRA have been made and approved for all changes outlined.

**Table 4:** Protocol changes made since the trial commenced

Aspect of trial	Changes made
Primary outcome point	Change from 3-months to 4-months. Due to family availability and referral rates, baseline interviews have been conducted over a longer time period than was anticipated. As a result, some families were being offered a 3-month follow-up appointment while still accessing the Young SMILES intervention (if randomised to that trial arm). To ensure the amount of ‘useful data’ is maximised, the primary outcome point was changed to 4 months, without altering the 6- and 12-month time points
Data collection	Addition of demographic questionnaire for all participants taking part in the feasibility trial (children and young people, parents, carers) Addition of referrer interviews to explore the experiences of individuals who have referred to the study and also those who have been approached but have not referred any families; individuals will be from health, social care, educational and third sector services, which will aid a more thorough understanding of the development of referral pathways that will add to the other findings from the feasibility study to assist with the development of an application for a full-scale trial
Randomisation	Change from using the Sealed Envelope system (www.sealedenvelope.com) to randomise 60 families on a 1:1 ratio either to Young SMILES or to usual care to online randomisation software (www.randomization.com), which would better meet study requirements in relation to sample size and the ability to stratify by age and site, without incurring costs Addition of 2:1 randomisation allocation procedures (2 Young SMILES intervention; 1 control) to expedite start of intervention groups; this has been acknowledged to be wholly appropriate for a feasibility trial in such a ‘specified group’ intervention
Intervention delivery	Inclusion of siblings within the same group (if in the same age band) Submission of young person travel consent form for parents/carers to consent to child/adolescent travelling to the intervention venue alone
Participant communication	Submission of letters/documents to communicate with families/sites during the trial, e.g. if unable to contact family or if any participant wishes to withdraw

## Discussion

The trial outlined is being conducted to explore if a structured, 8-week session-based intervention to improve the HRQoL in COPMI is feasible and acceptable. Once complete, it will offer valuable and timely insights into the delivery of evidence-based service development for this vulnerable population. Some barriers have been identified which may affect recruitment and completion of the study within existing timelines.

### Ethics and HRA issues

New REC/HRA approval processes delayed final REC/HRA approval in Phase I by 5 months. This, and the request that a separate application be submitted for Phase II, had a significant effect upon the start date of the feasibility trial. With final approvals for Phase II being delayed until June 2017, this resulted in an additional 3-month delay. Additional applications for one of our third sector partners following REC/HRA approval led to further unanticipated delays.

Once all approvals were in place, practitioners involved in the trial informed us that running groups during school holidays would be extremely challenging and have a significant and negative effect on their ability to engage families and CYP. Despite this, we worked with one site to pilot the feasibility of starting to run a group in the school summer holidays; but we were unable to identify any families during this time period, confirming the anticipated difficulties.

### Recruitment sites

Eight regional NSPCC service centres were closed as part of a strategic restructuring review prior to study recruitment start dates. Included in the closures was a local service centre we had planned to work with. This meant that we had to engage with two new NSPCC recruitment sites. This was achieved by the research team liaising with both sites to address queries and to establish new recruitment pathways. Changes in service management in one site have added to the workload and to the challenges of implementation of the Young SMILES at that site. The additional workload and greater distance from Manchester (compared to the original site, which was close to the research centre) has not only lengthened the time needed to complete the study tasks and delayed our milestones, but has also had a major effect on the budget allocated to travel (to deliver training, attend meetings, conduct data collection interviews with families).

Overall, the project timelines have been delayed by around 10-months by unforeseen events and procedural obstacles, which have been out of the control of the research team.

### Trial status

We are currently in the final stages of recruiting participants.

## Additional file

Additional file 1: SPIRIT 2013 Checklist: Recommended items to address in a clinical trial protocol and related documents. (DOC 132 kb)

## Abbreviations

CAMHS: Child and Adolescent Mental Health Services
CHU: Child Health Utility
COPMI: children and young people of parents with mental illness
CYP: children and young people
GP: general practitioner
HRA: Health Research Authority
HRQoL: health-related quality of life
NHS: National Health Service
NSPCC: National Society for the Prevention of Cruelty for Children
QALYs: Quality adjusted life years
RCT: randomised controlled trial
SMI: serious mental illness
SMILES: Simplifying Mental Illness + Life Enhancement Skills
TSC: Trial Steering Committee
